# The RAF Kinase Inhibitor Protein (RKIP): Good as Tumour Suppressor, Bad for the Heart

**DOI:** 10.3390/cells11040654

**Published:** 2022-02-14

**Authors:** Joshua Abd Alla, Ursula Quitterer

**Affiliations:** 1 Molecular Pharmacology, Department of Chemistry and Applied Biosciences, ETH Zurich, Winterthurerstrasse 190, 8057 Zurich, Switzerland; joshua.abdalla@pharma.ethz.ch; 2 Department of Medicine, Institute of Pharmacology and Toxicology, University of Zurich, Winterthurerstrasse 190, 8057 Zurich, Switzerland

**Keywords:** RKIP (RAF kinase inhibitor protein), *PEBP1* (phosphatidylethanolamine-binding protein 1), *RAF1*, *GRK2* (G protein-coupled receptor kinase 2), *AGTR1* (angiotensin II receptor type 1), *MAPK* (mitogen-activated protein kinase), tumour suppressor, heart failure, fibrosis

## Abstract

The RAF kinase inhibitor protein, RKIP, is a dual inhibitor of the RAF1 kinase and the G protein-coupled receptor kinase 2, GRK2. By inhibition of the RAF1-MAPK (mitogen-activated protein kinase) pathway, RKIP acts as a beneficial tumour suppressor. By inhibition of GRK2, RKIP counteracts GRK2-mediated desensitisation of G protein-coupled receptor (GPCR) signalling. GRK2 inhibition is considered to be cardioprotective under conditions of exaggerated GRK2 activity such as heart failure. However, cardioprotective GRK2 inhibition and pro-survival RAF1-MAPK pathway inhibition counteract each other, because inhibition of the pro-survival RAF1-MAPK cascade is detrimental for the heart. Therefore, the question arises, what is the net effect of these apparently divergent functions of RKIP in vivo? The available data show that, on one hand, GRK2 inhibition promotes cardioprotective signalling in isolated cardiomyocytes. On the other hand, inhibition of the pro-survival RAF1-MAPK pathway by RKIP deteriorates cardiomyocyte viability. In agreement with cardiotoxic effects, endogenous RKIP promotes cardiac fibrosis under conditions of cardiac stress, and transgenic RKIP induces heart dysfunction. Supported by next-generation sequencing (NGS) data of the RKIP-induced cardiac transcriptome, this review provides an overview of different RKIP functions and explains how beneficial GRK2 inhibition can go awry by RAF1-MAPK pathway inhibition. Based on RKIP studies, requirements for the development of a cardioprotective GRK2 inhibitor are deduced.

## 1. Introduction

The phosphatidylethanolamine-binding protein 1 (*PEBP1*) inhibits tumour metastasis initiation and acts as an endogenous tumour suppressor [1,2]. Based on its activity to inhibit the RAF1 kinase, this protein was renamed RAF kinase inhibitor protein, RKIP [3]. After the initial discovery as RAF1 kinase inhibitor, a panoply of different studies documented various functions of RKIP, which contribute to its role as an endogenous tumour suppressor [1,2,4,5,6]. RKIP is widely expressed in different tissues and does not only inhibit the RAF1 kinase but also interferes with many other growth-promoting pathways [1,2,3,4,5,6]. In addition to its tumour suppressor functions, research studies also focused on effects of RKIP in the heart. The underlying reason is the fact that all currently approved inhibitors of the RAF-MEK-ERK pathway have cardiotoxic side effects due to their apoptosis-enhancing activities and inhibition of the pro-survival RAF-MAPK pathway signalling [7,8,9]. The RAF1 kinase inhibitor RKIP is different from the available RAF-MEK-ERK inhibitors, because RKIP also acts as an endogenous inhibitor of the G protein-coupled receptor kinase 2, GRK2 [10]. By inhibition of the cardiac GRK2, proapoptotic signalling could eventually be counteracted by RKIP, because GRK2 inhibition confers cardio-protection, which is, in part, mediated by stimulation of the pro-survival RAF1-MAPK pathway of the heart [11].

In this context, RKIP-mediated inhibition of GRK2 is another area of research. GRK2 inhibition is an emerging treatment approach of heart failure. GRK2 is upregulated in failing hearts of patients with heart failure [12,13,14]. Inhibition of pathologically elevated GRK2 by different approaches, gene transfer and gene knockout showed beneficial effects in many models of cardiac dysfunction and heart failure [15,16,17]. GRK2 inhibition restrains the pathological GRK2 activity of failing hearts. The increased GRK2 leads to hyperphosphorylation of cardiac beta-adrenoceptors and deteriorates the impaired inotropic reserve of failing hearts. Apart from beta-adrenoceptor and GPCR (G protein-coupled receptor) phosphorylation, many nonreceptor substrates of GRK2 were identified in recent years, whose inhibition also contributes to beneficial actions of GRK2 inhibitors [18,19]. Based on its activity as a GRK2 inhibitor, it was proposed that RKIP could also exert beneficial, cardiac effects in vivo [20]. In contrast, several studies showed that potential cardioprotective activities of RKIP were counteracted in vivo by RKIP-mediated inhibition of the pro-survival RAF1-MAPK pathway and/or GRK2 inhibition-induced sensitisation of heart failure-promoting GPCRs such as the angiotensin II receptor type 1 (AGTR1), which impairs cardiac function [21,22,23]. This review gives an overview of the ambivalent nature of RKIP in vivo due to its dual functions as a RAF1 inhibitor and GRK2 inhibitor.

## 2. Tumour Suppressor Functions of RKIP

### 2.1. Discovery of RKIP as an Inhibitor of the RAF1 Kinase

The first RAF kinase, the v-Raf, was discovered in 1983 [24]. The gene was isolated from a murine retrovirus and named virus-induced rapidly accelerated fibrosarcoma (v-Raf) based on its capacity to transform fibroblasts into cancer-like cells [24]. Soon after the initial discovery, the cellular homologue of v-Raf was found as c-Raf [25,26,27], and the serine-threonine kinase activity of Raf was elucidated [28]. RAF1 is a central part of the first described mitogen-activated protein kinase (MAPK) pathway composed of RAF1-MEK-ERK1/2 [29,30,31]. Activation of this pathway occurs by Ras GTPases, which interact with RAF1 [32,33,34]. Other members of the RAF kinase family are ARAF and BRAF [35,36,37]. As part of the MAPK pathway, RAF1 acts as a mitogen-activated protein kinase kinase kinase, MAPKKK, which phosphorylates the mitogen-activated protein kinase kinase (MAPKK). In the classical MAPK pathway, MEK1 (*MAP2K1*) and MEK2 (*MAP2K2*) are the MAPKK. Upon activation by RAF1, MEK1 and MEK2 phosphorylate the MAPKs, ERK1 (*MAPK3*) and ERK2 (*MAPK1*) [38].

The *Raf1* kinase is indispensable for embryonic growth and differentiation, and knockout mice are not viable [39]. *Raf1*-deficient (*Raf1*−/−) mice die around mid-gestation due to enhanced apoptosis, which causes major defects of the placenta and the liver [39,40]. The apoptosis–inhibitory functions of RAF1 appear to be largely independent of MEK1/2 and ERK1/2. Apoptosis-inhibitory functions triggered by RAF1 are multiple and involve, e.g., (i) the interaction with the apoptosis-enhancing ASK1 (apoptosis signal-regulating kinase 1) [41], (ii) the BCL2 apoptosis regulator, which targets RAF1 to mitochondria to phosphorylate BAD (BCL2-associated agonist of cell death) and disrupt the BAD-BCL2 interaction [42], and (iii) the suppression of the proapoptotic MST2 kinase (STK3, serine/threonine kinase 3) [43].

The major pathologic interest in members of the RAF-MAPK pathway stems from numerous observations that this pathway is overactivated in 30% of all cancers [44]. Activating mutations of Ras GTPases, which are the upstream activators of RAF1, occur in about 19% of human neoplasms [45], and *BRAF* is mutated in about 7% of all cancers [46]. Among different malignancies, malignant melanoma has the highest incidence of oncogenic *BRAF* mutations with 27–70% [47]. Thus, the major oncogenic driver of the RAF family is BRAF. The frequency of activating *RAF1* mutations in human cancers is much lower, because RAF1 has a lower basal kinase activity than BRAF [48]. In agreement with this notion, *RAF1* mutations were detected in about 0.7% of human cancer cell lines [48]. Nevertheless, several types of human cancers show activating *RAF1* mutations [49,50]. For instance, there is a high frequency of *RAF1* gene rearrangements in pancreatic acinar cell carcinoma of 14.3–18.5% [49,50].

Based on the oncogenic potential of BRAF and RAF1 kinases, a large interest was and still is focused on the development of inhibitors of oncogenic activities of RAF kinase family members. The development of BRAF inhibitors and other MAPK pathway inhibitors was successful. The currently approved BRAF-MAPK pathway inhibitors are a mainstay of anticancer therapies for metastatic melanoma and other neoplastic diseases with activating and oncogenic MAPK pathway mutations [51,52]. In the framework of these efforts to develop an inhibitor of proto-oncogenic RAF kinases, a landmark study by Yeung et al., which was published in 1999 [3], identified the function of PEBP1 (phosphatidylethanolamine-binding protein 1) as an inhibitor of the RAF1 kinase. PEBP1 was found to interact with RAF1 in a yeast two-hybrid screen [3]. The inhibition of the RAF1 kinase activity by PEBP1 impairs the activating MEK1 phosphorylation by RAF1 [3,53]. Based on its RAF1 kinase-inhibitory function, PEBP1 was renamed RAF kinase inhibitor protein, RKIP [3]. Subsequent studies confirmed the function of RKIP as RAF1 inhibitor [53,54]. It was found that RKIP, depending on the cellular background, not only inhibits RAF1 but also BRAF [55,56,57]. 

The activity of RKIP as a RAF1 inhibitor is regulated by protein kinase C (PKC)-mediated phosphorylation of RKIP on serine-153 [10,58]. This phosphorylation leads to a dissociation of RKIP from RAF1 [10,58]. RKIP dissociation from RAF1 leads to an enhanced RAF1-mediated kinase activity and contributes to the PKC-induced enhancement of RAF1-mediated signalling [58]. Taken together, the PEBP1 alias RKIP acts as a RAF kinase inhibitor protein, which is regulated by PKC phosphorylation.

### 2.2. Apoptosis-Enhancing Functions of RKIP

RKIP is a ubiquitously expressed protein with multiple functions. As inhibitor of the pro-survival RAF-MAPK pathway, RKIP acts as an endogenous tumour suppressor. A major RKIP-mediated tumour suppressive function is based on its enhancement of tumour cell death and apoptosis. Proapoptotic activities of RKIP involve the inhibition of RAF1 together with other mechanisms. By the inhibition of RAF1, RKIP prevents the translocation of RAF1 to mitochondria [59]. Mitochondrial translocation of RAF1 contributes to antiapoptotic effects of RAF1 [42]. Consequently, inhibition of mitochondrial RAF1 translocation by RKIP prevents antiapoptotic RAF1 signalling and enhances the cellular sensitivity to apoptosis [59].

In addition to RAF1 kinase inhibition, RKIP interferes with other growth-promoting pathways and enhances proapoptotic stimuli. Among several important signalling pathways, RKIP interferes with NF-kappa B signalling by the inhibition of upstream kinases TAK1 (transforming growth factor-beta-activated kinase 1; MAP3K7), NIK (NF-kappa beta-inducing kinase; MAP3K14) and IKKA and IKKB (inhibitor of nuclear factor kappa B kinase subunit alpha and beta; IKBKA, and IKBKB) [60]. The net result is inhibition of the transcription factor NF-kappa B complex by a decrease of IKBA (NF-kappa B inhibitor alpha; NFKBIA) phosphorylation and prevention of IKBA degradation. By this mechanism, RKIP inhibits antiapoptotic genes and promotes the de-repression of proapoptotic genes such as the death receptors [60]. As a consequence, RKIP sensitises cells to apoptosis. This activity of RKIP is synergistic with inhibition of RAF1-mediated effects, which also contributes to enhanced cellular sensitivity to apoptosis.

Another apoptosis-enhancing function is attributed to the interaction of RKIP with GSK3B (glycogen synthase kinase 3 beta), which is a suppressor of many oncogenic signalling cascades. RKIP enhances the suppressive functions of GSK3B by interaction with GSK3B and reduction of inhibitory GSK3B phosphorylation on T390 by P38 MAP kinase (MAPK14) [61]. By this mechanism, RKIP could positively modulate GSK3B activity in cells, in which GSK3B exerts tumour suppressive and apoptosis-stimulatory functions [62].

Finally, RKIP was also reported to de-repress the expression of death receptors (DR) such as DR5 [63]. By this mechanism RKIP sensitises tumour cells to apoptosis triggered by the TRAIL (tumour necrosis factor-related apoptosis-inducing ligand) and Fas ligand [63]. Taken together, by inhibition of RAF1 and other antiapoptotic stimuli, RKIP enhances cancer cell apoptosis directly and/or abrogates tumour cell resistance to apoptosis-enhancing stimuli.

### 2.3. Metastasis-Suppressing Functions of RKIP

One of the main functions of RKIP is metastasis suppression, which is mediated by multiple mechanisms. A major pathway of RKIP-dependent metastasis suppression involves the inhibition of MYC. Since MYC is a direct target of the RAF1-MEK1/2-ERK1/2 cascade, RKIP-mediated metastasis suppression by MYC inhibition is a direct consequence of RAF1 inhibition. By inhibition of the RAF1-MAPK pathway, RKIP decreases ERK1/2-mediated MYC phosphorylation and activation. As a consequence, MYC-mediated LIN28 expression is repressed by RKIP. In a next step, downregulation of LIN28 by RKIP de-represses let-7 [64]. Within a complex tumour metastasis-enhancing network, the final result is that RKIP induces let-7, which is known to inhibit HMGA2 (high mobility group AT-hook 2) [65,66]. The expression of HMGA2 is further suppressed by RKIP via induction of the microRNA, miR-185 [67]. Inhibition of HMGA2 blocks SNAIL1 (SNAI1) transcription and other mechanisms involved in tumour cell invasion and metastasis [68,69]. Taken together, RKIP interferes with tumour cell invasiveness and metastasis by induction of let-7 [64,65].

By inhibition of the NF-kappa B signalling pathway, RKIP also interferes with early metastasis and the induction of epithelial–mesenchymal transition, EMT [60]. EMT is a major step in cancer metastasis and accounts for the loss of cell-cell contacts and tumour cell dissemination within the body [70,71,72]. Several studies have documented that RKIP mediates reversal of the EMT transformation of cancer cells by various mechanisms [73,74,75,76]. Reversal of EMT transformation by RKIP could involve the inhibition of NOTCH1-Notch1 signalling by decreasing the Notch1 intracellular domain, NICD, which is involved in EMT and metastasis [76].

**Figure 1:**
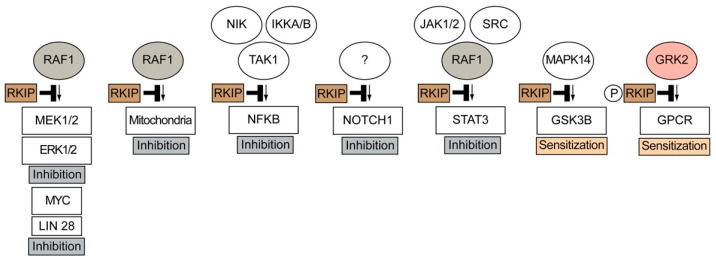
Overview of RKIP-mediated functions induced by inhibition of RAF1, GRK2 and other kinases. RKIP-mediated kinase inhibition leads to signal inhibition and signal sensitisation. Kinase inhibition by RKIP (RAF1 kinase inhibitor protein) leads to inhibition of the RAF1-MEK1/2-ERK1/2 pathway and NFKB, NOTCH1 and STAT3 pathways. By inhibition of P38 (MAPK14), RKIP enhances GSK3B activity, and RKIP-mediated inhibition of GRK2 sensitises signalling stimulated by G protein-coupled receptors, GPCRs (NIK, NF-kappa beta-inducing kinase; IKKA and IKKB, inhibitor of nuclear factor kappa B kinase subunit alpha and beta; TAK1, transforming growth factor-beta-activated kinase 1; JAK1/2, Janus kinase 1 and 2; STAT3, signal transducer and activator of transcription 3; GSK3B, glycogen synthase kinase 3 beta; MAPK14, mitogen-activated protein kinase 14, P38 MAP kinase; GRK2, G protein-coupled receptor kinase 2).

Another metastasis-suppressing activity of RKIP involves inhibition of the STAT3 (signal transducer and activator of transcription 3) signalling pathway [77,78]. RKIP may also inhibit metastasis by inhibition of MMPs (matrix metalloproteinases), e.g., RKIP inhibits breast cancer invasion by preventing the transcription of MMP13 [79]. This activity is also due to inhibition of the RAF1-MEK1/2-ERK1/2 pathway [79]. Finally, RKIP regulates the tumour microenvironment and blocks the recruitment of metastasis-enhancing tumour-associated macrophages, TAMs [80,81]. Taken together, RKIP is a kinase inhibitor and metastasis suppressor, which inhibits tumour cell invasiveness by inhibition of RAF1 and interference with other tumour-promoting pathways (Figure 1). Kinase inhibition by RKIP not only results in signal inhibition but also accounts for signal sensitisation (Figure 1). 

### 2.4. The Endogenous Tumour Metastasis Suppressor, RKIP, Is Frequently Downregulated in Metastatic Tumours

Based on cellular and mechanistic data, many researchers have investigated whether RKIP could also act as a tumour suppressor in vivo. On one hand, experimental data document that overexpression of RKIP blocks proliferation and/or metastatic progression of various experimental tumour models in vivo [67,82,83,84]. On the other hand, RKIP is regularly downregulated in human metastatic tumours, e.g., References [85,86,87,88,89,90]. Downregulation of RKIP in tumours is caused by different mechanisms, which vary among tumours and tumour cells [85,86,87,88,89,90]. RKIP downregulation can be mediated by epigenetic mechanisms such as promoter methylation [85,86,87,88] and/or histone modification [88]. Promoter methylation was reported to cause RKIP downregulation in many different tumours and tumour cells, e.g., oesophageal squamous cell carcinomas, gastric adenocarcinomas and breast carcinoma cells [86,87,88]. In some tumour cells such as triple-negative breast carcinoma cells, different epigenetic mechanisms, i.e., promoter methylation and histone modification, additively contribute to RKIP downregulation [88]. Another well-established mode of RKIP silencing involves negative transcriptional regulation due to the direct binding of transcription factors and co-factors to the RKIP promoter [89,90]. Transcriptional repressor SNAI1 (snail family transcriptional repressor 1) and transcription factor BACH1 (BTB and CNC homolog 1) were identified as negative regulators of RKIP expression [89,90]. In contrast, by binding to cis-acting elements within the RKIP promoter region, some transcription factors also act as positive regulators and enhance RKIP transcription, such as CREB1 (cAMP-responsive element-binding protein 1), SP1 (specificity protein 1) and EP300 (E1A binding protein P300) [91]. In addition to transcriptional regulation, post-transcriptional silencing of RKIP by selected microRNAs was also reported [74,92]. Finally, the RKIP protein levels are regulated at the post-translational level, e.g., by targeting of the RKIP protein to proteasomal degradation. Enhanced proteasome-mediated degradation of non-phosphorylated RKIP occurred in gastric cancer cells and was stimulated by *H. pylori* infection [93].

In sum, RKIP is an endogenous tumour metastasis suppressor that is widely expressed in non-tumour cells. However, as an endogenous tumour suppressor, this protein is regularly downregulated within the tumour environment. The major functions of RKIP as a tumour suppressor are mediated by its main activity as an inhibitor of the proto-oncogenic and pro-survival RAF1-MAPK pathway (Figure 1).

## 3. Inhibition of GRK2 by RKIP

RKIP not only inhibits the RAF1 kinase but also the G protein-coupled receptor kinase, GRK2 (Figure 1 and Reference [10]). GRK2 is a member of the GRK family, which is composed of seven isoforms, GRK1–GRK7 [94]. By phosphorylation of activated G protein-coupled receptors, GRKs initiate the process of signal desensitisation and receptor downregulation [94]. GRK2 is the first nonvisual receptor kinase that was elucidated [94,95]. GRK2 is of great physiological importance and exerts indispensable functions, like the RAF1 kinase. Deficiency of GRK2 is lethal [96,97]. Grk2 knockout mice died on day 15 of embryonic development from cardiac hypoplasia and cardiac dysfunction [96]. The pathophysiological relevance of GRK2 has been documented by many studies. For instance, an exaggerated GRK2 activity contributes to symptoms of cardiovascular diseases such as heart failure and hypertension [12,98,99,100,101]. In addition, an increased GRK2 activity is involved in a variety of other pathologies, e.g., diabetes, inflammation and osteoporosis [102,103,104,105,106]. Under conditions with pathologically increased GRK2 activity, the inhibition of elevated GRK2 activity is beneficial [98,99,102,103,104,105,106]. Therefore, the search and development of GRK2 inhibitors is a wide area of research. In this context, RKIP was identified as a GRK2 inhibitor, which blocks detrimental GRK2-mediated signalling in isolated cardiomyocytes [10].

**Figure 2:**
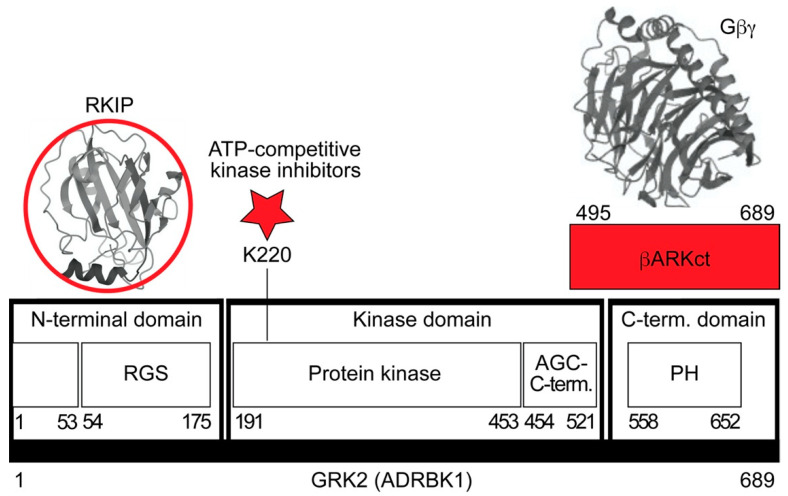
Schematic overview of GRK2 (G protein-coupled receptor kinase) and possible approaches to GRK2 inhibition. GRK2 consists of three domains, the RGS (regulator of G protein signalling)-containing N-terminal domain, the kinase domain and the C-terminal domain with the PH (pleckstrin homology) domain. The βARKct consists of amino acids 495–689 of GRK2 and scavenges Gβγ subunits (structure of Gβ1γ2 extracted from: PDB 4MK0), which are required for GRK2 activation and membrane translocation. ATP-competitive kinase inhibitors target the binding of ATP to the ATP-binding site at K220. RKIP (structure from: PDB 2QYQ) interacts with the GRK2 N-terminal domain containing a receptor binding site and the RGS domain. GRK2 inhibitors are highlighted in red.

How does RKIP inhibit GRK2? There are different approaches of GRK2 inhibition (Figure 2). 

The most straightforward approach would be the inhibition of the GRK2 kinase activity by an ATP-site directed kinase inhibitor (Figure 2). However, GRK2 is a member of the GRK family with several closely related kinases, and approaches to develop GRK2 subtype-selective kinase inhibitors for patient use have been so far unsuccessful [107,108]. By drug repurposing, the antidepressant drug and selective serotonin reuptake inhibitor (SSRI) paroxetine was identified as an ATP-competitive GRK2 inhibitor [109]. In experimental models of cardiac dysfunction, paroxetine showed cardioprotective activities, which could be attributed to GRK2 inhibition [109].

Due to the difficulty to develop a GRK2-specific small molecule inhibitor, other approaches of GRK2 inhibition were embarked. Those approaches of GRK2 inhibition apply proteins to disrupt protein–protein interactions. In this context, a frequently used strategy of GRK2 inhibition targets the membrane translocation and activation of GRK2 by Gβγ subunits of heterotrimeric G proteins (Figure 2). Following this concept, the C-terminal Gβγ-binding pleckstrin homology (PH) domain of GRK2 (βARKct) was identified as a GRK2 inhibitor [106]. GRK2 inhibition by βARKct is successful in vivo and could improve the disease symptoms of heart failure in numerous experimental cardiovascular disease models of mouse, rat, rabbit and porcine origins [15,110,111,112]. In contrast to βARKct, RKIP inhibits GRK2 by shielding of the N-terminal domain of GRK2 (Figure 2 and Reference [10]). The extreme N-terminal domain of GRK2 is required for receptor interaction and enhancement of the catalytic activity of GRK2 to phosphorylate receptor substrates [113]. Consequently, by interaction with the GRK2 N-terminal domain, RKIP selectively inhibits the phosphorylation of receptor substrates by GRK2, whereas nonreceptor substrates of GRK2 are not affected by RKIP [10]. The interaction of RKIP with GRK2 requires phosphorylation of RKIP on serine-153 by protein kinase C, PKC [10]. PKC-mediated phosphorylation on serine-153 dissociates RKIP from RAF1 and switches RKIP from RAF1 to GRK2 [10,58]. Among different GRK family members, RKIP specifically interacts with GRK2 but has little activity towards GRK5, which is a member of the GRK4 subfamily consisting of GRK4, GRK5 and GRK6 [10].

RKIP-mediated inhibition of GRK2 and ensuing signal enhancement of various G-protein-coupled receptors was documented in several studies [114,115]. Notably, the highly expressed RKIP in the nervous system sensitises signalling stimulated by different opioid receptors [114,115,116]. The underlying mechanism involves the bradykinin B2 receptor-induced activation of PKC, which promotes RKIP phosphorylation on serine-153 and GRK2 inhibition. As a result, bradykinin sensitises mu-opioid receptor and delta-opioid receptor signalling [114,115].

By GRK2 inhibition, RKIP prevents GRK2-mediated phosphorylation and desensitisation of β-adrenergic receptors [10]. β-adrenergic receptors are the prototypical GPCR substrates of GRK2, which was therefore initially named β-adrenergic receptor kinase 1, ADRBK1 [94,95]. RKIP inhibits Grk2 in isolated rat cardiomyocytes, and endogenously expressed RKIP levels are sufficient to dampen Grk2-mediated desensitisation of β-adrenoceptors in cardiomyocytes [10]. By this mechanism, RKIP enhances the β-adrenoceptor-stimulated cardiomyocyte beating frequency [10]. Taken together, RKIP acts as an endogenous GRK2 inhibitor, which specifically blocks the GRK2-mediated phosphorylation of receptor substrates. Since different approaches of GRK2 inhibition show cardio-protection in vivo, there was a great interest to investigate whether RKIP-mediated GRK2 inhibition could be exploited to develop an RKIP-based cardioprotective therapy.

## 4. RKIP Causes Symptoms of Heart Failure In Vivo

### 4.1. RKIP Promotes Features of Heart Failure by Stimulation of Hypertrophic and Apoptotic Signalling

**Figure 3:**
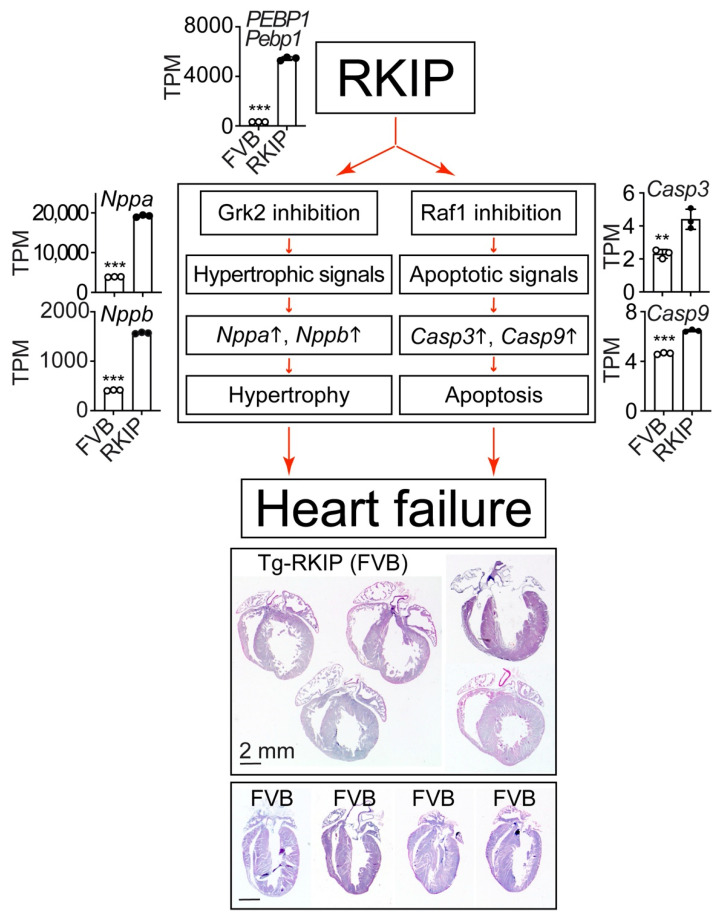
RKIP promotes features of heart failure by stimulation of hypertrophic and apoptotic signalling. The dual functions of RKIP (RAF kinase inhibitor protein) as Grk2 and Raf1 inhibitor promote hypertrophic and apoptotic signalling with significantly increased transcript levels of hypertrophic markers *Nppa* and *Nppb* (natriuretic peptide A and B) and apoptosis-promoting executioner caspase 3 (*Casp3*) and initiator caspase 9 (*Casp9*) in Tg-RKIP hearts with 16.74 ± 0.44-fold increased *PEBP1*-*Pebp1* (phosphatidylethanolamine-binding protein 1) transcript levels compared to those of FVB controls. The transcript levels are presented as transcripts per million, TPM (mean ± s.d.; ***, *p* < 0.0001; **, *p* = 0.0055; unpaired, two-tailed *t*-test). Lower panels: haematoxylin–eosin-stained cardiac specimens of 8-month-old, male Tg-RKIP mice (*n* = 5) document the phenotype of dilated cardiomyopathy induced by RKIP in comparison to age-matched, male, nontransgenic FVB mice (*n* = 4). Data come from our published studies of Tg-RKIP mice with FVB background [11,22]. NGS data are available at the NCBI GEO database, accession number GSE191316.

RKIP-mediated inhibition of GRK2 was initially characterised in cell lines and isolated cardiomyocytes [10]. Therefore, additional research studies were conducted, which investigated the cardiac phenotype induced by RKIP in vivo. Elucidation of the in vivo function of RKIP has to consider the dual activities of RKIP as a GRK2 inhibitor and RAF1 inhibitor (Figure 3). 

On one hand, numerous studies documented that GRK2 inhibition is cardioprotective [12,98,99,100,101,111,112]. On the other hand, inhibition of Raf1 by gene knockout in mice is detrimental for the heart, enhances cardiomyocyte apoptosis and promotes heart dilatation and cardiac dysfunction [117,118]. Clinical data with inhibitors of the MAPK pathway components, BRAF, MEK1/2 and ERK1/2, which are approved as anticancer therapeutics, confirmed the cardiotoxic side effects of RAF-MAPK pathway inhibition in humans [7,8,9,119].

To investigate how the heart reacts to these apparently opposing functions of RKIP in vivo, the most straightforward approach was the generation of RKIP-transgenic mice with myocardium-specific expression of RKIP under control of the alpha-MHC-promoter [11,21,22]. Different transgenic mouse lines were generated in different genetic backgrounds [11,21,22]. 

Phenotyping studies revealed that moderately increased RKIP protein levels in the heart led to enhanced cardiomyocyte apoptosis, induced features of cardiomyocyte hypertrophy and caused dilated cardiomyopathy with cardiac dysfunction in mice with B6 or FVB background [11,21,22]. Symptoms of heart failure of RKIP-transgenic mice were documented by a significantly decreased left ventricular ejection fraction, which was determined by echocardiography [11,21,22]. A histological assessment of paraffin-embedded heart specimens of Tg-RKIP mice showed a phenotype of ventricular dilation and cardiomyocyte enlargement [11,21,22].

RKIP induces heart failure by its dual functions as a GRK2 inhibitor and RAF1 inhibitor (Figure 3). By inhibition of GRK2, RKIP promotes stimulation of hypertrophic signalling, which is partially attributed to sensitisation of the angiotensin II receptor type 1, Agtr1 (22). By inhibition of Raf1, RKIP enhances apoptotic signalling and cardiomyocyte apoptosis (11). Hypertrophic signalling is documented by increased cardiac transcript levels of natriuretic peptides A (Nppa) and B (Nppb), and apoptotic signalling is documented by increased levels of the executioner caspase 3, Casp3, and the initiator caspase 9, Casp9 (Figure 3).

The cardiac phenotype of Tg-RKIP mice with cardiac dysfunction, cardiomyocyte apoptosis and dilated cardiomyopathy reproduces major hallmarks of the phenotype triggered by inhibition of the RAF(1)-MAPK pathway [7,8,9,117,118,119]. Taken together, Tg-RKIP mice with myocardium-specific expression of the dual RAF1 and GRK2 inhibitor, RKIP, developed a phenotype of dilated cardiomyopathy with features of cardiomyocyte hypertrophy and cardiomyocyte apoptosis [11,21,22].

### 4.2. RAF1-MAPK Pathway Inhibition by RKIP Causes Upregulation of Heart Failure-Promoting Lipid Metabolism Genes

**Figure 4:**
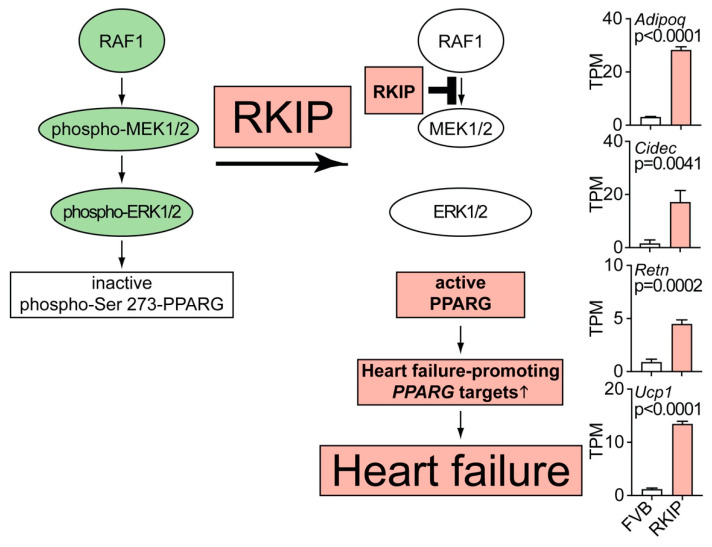
RAF1-MAPK pathway inhibition by RKIP causes the upregulation of heart failure-promoting lipid metabolism genes. The scheme illustrates how RKIP-mediated inhibition of RAF1-MEK1/2-ERK1/2 promotes the activation of *PPARG* (peroxisome proliferator-activated receptor gamma) with ensuing upregulation of heart failure-promoting lipid metabolism genes and cardiolipotoxicity. Transcript levels of the selected *Pparg* target genes are increased in hearts of 8-month-old, male Tg-RKIP mice with FVB backgrounds compared to those of non-transgenic FVB mice (*n* = 3 hearts per group; mean ± s.d.). Transcript levels of adiponectin (*Adipoq*), the lipid droplet associated cell death-inducing DFFA-like effector protein c (*Cidec*), resistin (*Retn*) and the mitochondrial brown fat uncoupling protein 1 (*Ucp1*) are presented. *p*-values were determined by the unpaired, two-tailed *t*-test. Data come from our published studies of Tg-RKIP mice with FVB background [22]. NGS data are available at the NCBI GEO database, accession number GSE191316.

Does RKIP act as RAF1 inhibitor in vivo? This question was addressed in Tg-RKIP mice with B6 and FVB background. Data showed that RKIP caused myocardial dysfunction and cardiolipotoxicity [21,22]. The RKIP-induced cardiac lipid overload could be attributed to RKIP-mediated RAF1-MEK1/2-ERK1/2 inhibition (Figure 4). By inhibition of the RAF1-MAPK pathway, cardiac RKIP prevents the Erk1/2 (Mapk3/1)-dependent inhibitory phosphorylation of Pparg (peroxisome proliferator-activated receptor gamma) on serine-273 [21,22]. The RKIP-mediated reduction of Pparg serine-273 phosphorylation triggers Pparg-dependent lipid metabolism targets, which promote a cardiac lipid overload and cardiac dysfunction [21,22]. Some of those genes, which are specifically induced upon Pparg dephosphorylation on serine-273, are adiponectin (Adipoq), the lipid droplet associated cell death-inducing DFFA-like effector protein c (Cidec), resistin (Retn) and the mitochondrial brown fat uncoupling protein 1 (Ucp1) [120]. These Pparg target genes were induced in vivo by transgenic RKIP expression or by treatment with the heart failure-promoting Pparg agonist, rosiglitazone [21,22,121].

As documented by published studies, all of these RKIP-induced Pparg target genes promote symptoms of heart failure and/or cardiac lipid overload, i.e., Adipoq [122], Cidec [123], Retn [124] and Ucp1 [21]. Concomitantly with upregulation of these genes of the cardiac lipid metabolic process, Tg-RKIP mice developed symptoms of heart failure and cardiac lipid overload [21,22]. Thus, inhibition of the RAF1-MAPK axis by RKIP decreases the inhibitory Erk1/2-mediated phosphorylation of Pparg on serine-273. As a consequence, RKIP induces Pparg activation and induction of heart failure-promoting lipid metabolism genes (Figure 4). Inhibition of the pro-survival RAF1-MAPK pathway and cardiotoxic lipid overload both contribute to RKIP-enhanced cardiomyocyte apoptosis and the heart failure phenotype.

### 4.3. RKIP-Mediated GRK2 Inhibition Leads to Sensitisation of the Heart Failure-Promoting Angiotensin II Receptor Type 1 (AGTR1) and Cardiac Fibrosis

**Figure 5:**
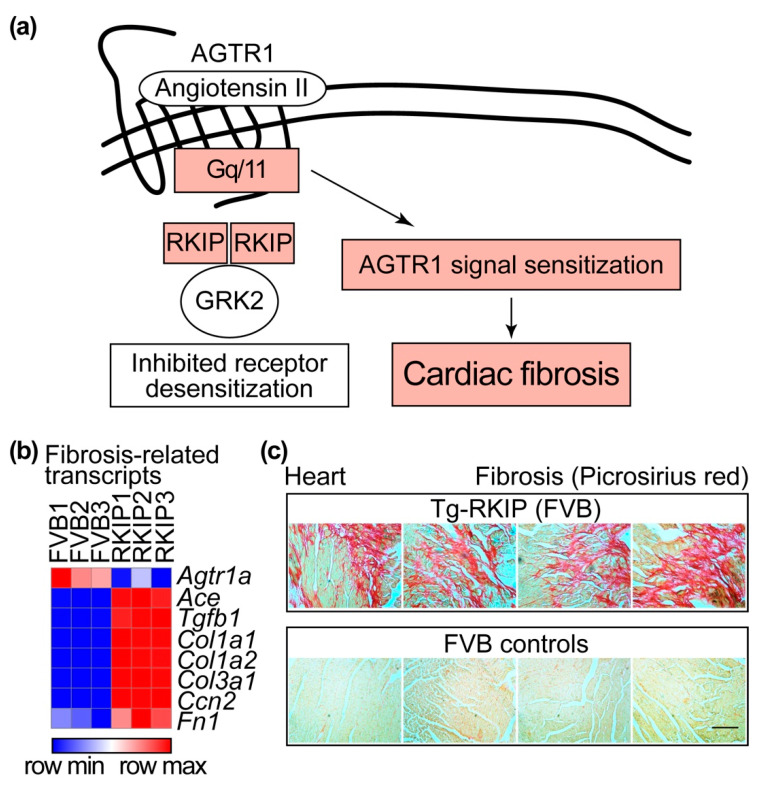
RKIP-mediated GRK2 inhibition leads to sensitisation of the heart failure-promoting AGTR1 and cardiac fibrosis. (**a**) Schematic illustration of the RKIP-mediated pathway leading to cardiac fibrosis. (**b**) Heat map of the relative cardiac transcript levels of fibrosis-related *Agtr1a* (angiotensin II receptor type 1a), *Ace* (angiotensin-converting enzyme), *Tgfb1* (transforming growth factor beta 1), *Col1a1* and *Col1a2* (collagen type I alpha 1 and 2 chain), *Col3a1* (collagen type III alpha 1 chain), *Ccn2* (cellular communication network factor 2) and *Fn1* (fibronectin 1) of Tg-RKIP mice and FVB controls (*n* = 3). (**c**) Cardiac fibrosis in 8-month-old male Tg-RKIP mice (upper panels) was detected by picrosirius red staining of cardiac specimens and compared to age-matched, non-transgenic FVB mice (lower panels), which were used as controls (*n* = 4 hearts per group; bar: 40 μm). Data come from our published studies of Tg-RKIP mice with FVB background [11,22]. NGS data are available at the NCBI GEO database, accession number GSE191316. The heat map was generated by Morpheus, https://software.broadinstitute.org/morpheus (accessed on 26 December 2021).

As detailed above, inhibition of the pro-survival RAF1-MAPK pathway by RKIP enhances the cardiotoxic lipid overload and cardiomyocyte apoptosis. However, inhibition of GRK2 by RKIP promotes cardioprotective signalling in cells and cardiomyocytes [10,20]. Why does RKIP not act as a cardioprotective GRK2 inhibitor in vivo and counteract the RAF1-MAPK pathway inhibition-induced cardiac dysfunction? This question is relevant, because GRK2 inhibition by many different approaches is cardioprotective in a panoply of different experimental models e.g., References [11,15,16,17,21,22]. Furthermore, GRK2 inhibition by different approaches is capable of counteracting the cardiotoxic lipid load induced by RAF1-MAPK pathway inhibition [21,22]. To answer this question, it needs to be considered that the mode of RKIP-mediated GRK2 inhibition involves the interaction with the N-terminal domain of GRK2 (cf. Figure 2). By interaction with the N-terminal domain of GRK2, RKIP disrupts the interaction of GRK2 with receptor substrates. As a consequence, RKIP specifically inhibits GRK2-mediated GPCR substrate phosphorylation and thereby prevents receptor desensitisation [10]. By this specific mode of GRK2 inhibition, RKIP promotes the sensitisation of signalling stimulated by GPCRs such as angiotensin II receptor type 1, AGTR1 (Figure 5a). Since AGTR1 promotes cardiac fibrosis and hypertrophy, RKIP sensitises profibrotic and hypertrophic signalling stimulated by AGTR1 [22,23].

The overstimulation of Agtr1 in Tg-RKIP mice with heart failure is reflected by the downregulation of Agtr1-specific binding sites [22] and cardiac *Agtr1a* transcript levels (Figure 5b). Decreased transcript and protein levels of AGTR1 are also a characteristic feature of failing human hearts [125,126]. The downregulation of AGTR1 is caused by overstimulation of AGTR1 with angiotensin II [127,128]. In Tg-RKIP hearts, the sensitisation and downregulation of *Agtr1a* are triggered by the GRK2-inhibitory function of RKIP (Figure 5). During the course of heart failure pathogenesis, overstimulation with the subsequent downregulation of *AGTR1* transcript levels is further enhanced by increased levels of the angiotensin II-generating, angiotensin-converting enzyme (*ACE*), which is elevated on biopsy specimens of failing human hearts [129,130] and RKIP-transgenic hearts (Figure 5b). 

The heart failure-promoting AGTR1-Agtr1 enhances myocardial fibrosis [131,132]. AGTR1-Agtr1-induced myocardial fibrosis is either directly mediated by the angiotensin II stimulation of AGTR1-Agtr1 [133] or indirectly by the AGTR1-Agtr1-mediated induction of transforming growth factor beta 1, *TGFB1*-*Tgfb1* [134]. The sensitisation of Agtr1 by RKIP promotes myocardial fibrosis [22,23]. Transcriptome profiling documented increased fibrosis marker genes in Tg-RKIP hearts, i.e., *Col1a1* and *Col1a2* (collagen type I alpha 1 and 2 chain), *Col3a1* (collagen type III alpha 1 chain), *Ccn2* (cellular communication network factor 2; *Ctgf*) and *Fn1* (fibronectin 1) (Figure 5b). All of these genes are upregulated by enhanced AGTR1-Agtr1 stimulation [121,135,136,137]. Myocardial fibrosis was confirmed by picrosirius red staining of heart specimens from Tg-RKIP mice (Figure 5c and Reference [22]).

Complementary studies with RKIP-deficient mice found that endogenous RKIP levels are sufficient to enhance myocardial fibrosis under conditions of enhanced myocardial stress imposed, e.g., by chronic pressure overload induced by transverse aortic constriction or carbon tetrachloride treatment [23]. Fibrosis-promoting functions of endogenously expressed RKIP were—at least partially—also attributed to the sensitisation of Agtr1 by RKIP and enhanced Agtr1-stimulated signalling [23]. As a consequence, RKIP decreases transcription of antioxidant and cytoprotective genes by preventing the nuclear localisation and activity of the antioxidant transcription factor nuclear factor erythroid-derived 2-like 2 (*Nfe2l2*; *Nrf2*) [23,138,139]. The ensuing raised ROS production contributes to RKIP-increased cell death and fibrosis [23].

The profibrotic activities of RKIP could be neutralised by a defective *Nnt* (nicotinamide nucleotide transhydrogenase) gene locus in the B6 C57BL/6J background whereas in the B6 C57BL/6N background, intact *Nnt* promotes mitochondrial ROS generation under chronic pressure overload [23,140]. Complementary to those studies, RKIP exerted profibrotic functions not only in B6 (C57BL/6N) background but also in FVB background [22,23]. 

Taken together, different studies with RKIP-transgenic mice and RKIP knockout mice demonstrate that RKIP accelerates and/or promotes symptoms of cardiac dysfunction, heart failure and cardiac fibrosis in vivo [11,21,22,23]. Heart failure-promoting activities of RKIP involve the dual functions of RKIP as a RAF1 inhibitor and GRK2 inhibitor. Several lines of evidence show that inhibition of the pro-survival RAF1-MAPK pathway by RKIP enhances cardiomyocyte death and promotes cardiotoxic lipid overload [11,21,22,23]. Inhibition of GRK2 by RKIP sensitises the heart failure-promoting AGTR1-Agtr1, which triggers cardiac fibrosis [22,23].

### 4.4. Effects of RKIP on Cardiac GRKs and β-Adrenoceptors 

During the pathogenesis of heart failure, there is an upregulation of *GRK2-Grk2*, which accounts for the impaired contractile reserve of failing hearts by desensitisation and downregulation of cardiac β-adrenoceptors [12,13,14,15,16,17,141]. Inhibition of the exaggerated GRK2-Grk2 activity by a GRK2 inhibitor or (partial) *Grk2* deficiency (*Adrbk1*+/−) in failing hearts restores the contractile response and contributes to the cardioprotective profile of GRK2-Grk2 inhibitors [13,14,15,16,17]. Upon improvement of the impaired cardiac function, the upregulated *GRK2-Grk2* levels are normalised [14,142]. Likewise, therapy with a cardioprotective GRK2 inhibitor, such as the βARKct, lowered the pathologically elevated *Grk2* levels of heart failure rats [143]. 

**Figure 6:**
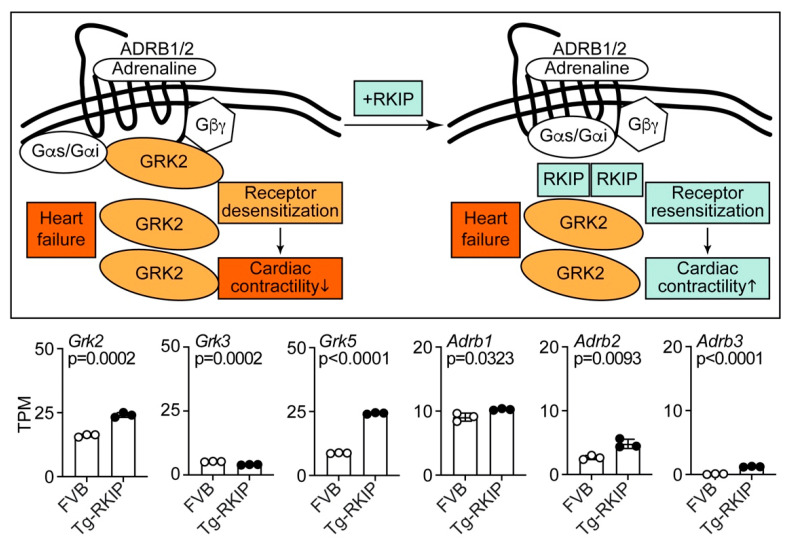
Effects of RKIP on cardiac *Grk* family members and β-adrenoceptors. The scheme illustrates effects of RKIP on GRK2 inhibition and β-adrenoceptor resensitisation in heart failure (upper right) versus the heart failure control (upper left). The lower part presents cardiac transcript levels of *Grk2*, *Grk3* and *Grk5* and beta-adrenoceptors *Adrb1*, *Adrb2* and *Adrb3* of eight-month-old, male Tg-RKIP mice and age-matched, non-transgenic, male FVB mice. Data come from our published studies of Tg-RKIP mice with FVB background [11,22]. Data are means ± s.d. (*n* = 3 mice/group). *p*-values are indicated and were determined by the unpaired, two-tailed *t*-test. NGS data are available at the NCBI GEO database, accession number GSE191316.

As an inhibitor of GRK2, RKIP also prevents GRK2-mediated desensitisation of β-adrenoceptors (Adrb1/2) and enhances the β-adrenoceptor-mediated contractile activity of cardiomyocytes (Figure 6, and [10,20]). However, in contrast to inhibition of GRK2 by the cardioprotective βARKct [143], Tg-RKIP mice showed an upregulation of the cardiac *Grk2* transcript levels in vivo, i.e., the *Grk2* transcript levels of Tg-RKIP hearts were 1.50 ± 0.06-fold higher than those of non-transgenic FVB controls (Figure 6).

Upregulation of *Grk2* is induced by long-term stimulation of β-adrenoceptors [142] and could reflect overstimulation of the sympathetic nervous system, which is characteristic of heart failure [14]. In this context, the upregulation of *Grk2* in Tg-RKIP hearts could indicate that the Grk2-inhibition-mediated sensitisation of β-adrenoceptors by RKIP could lead to *Grk2* upregulation. For comparison, the cardiac transcript levels of *Grk3* were not increased but slightly decreased in Tg-RKIP hearts with symptoms of heart failure (Figure 6). This observation complements previous data with a rat heart failure model in which *Grk3* also was not elevated [144]. *GRK5* is another member of the GRK family, which is upregulated in human patients with heart failure [145,146]. Tg-RKIP hearts showed a significant 2.78 ± 0.03-fold upregulation of the *Grk5* transcript levels (Figure 6). Increased cardiac *Grk5* levels in Tg-RKIP mice are detrimental, because *Grk5* contributes to pathological cardiac hypertrophy and Agtr1-stimulated fibrosis [147,148]. The upregulation of *Grk5* in failing hearts is independent of β-adrenoceptor stimulation [142]. Taken together, the hearts of Tg-RKIP mice show upregulation of *Grk2* and *Grk5* and thereby resemble heart specimens of human patients with heart failure [145,146]. 

While *GRK2* is upregulated, several studies documented that the β1-adrenoceptor (*ADRB1*) is downregulated in human heart failure [12,141,149,150]. The downregulation of *ADRB1* is considered to involve the exaggerated GRK2 activity of failing hearts [12,141,149,150]. In contrast, Tg-RKIP mice with heart failure did not show a downregulation of the *Adrb1* transcript levels (Figure 6). Instead, *Adrb1* was slightly increased in Tg-RKIP hearts (Figure 6). The absent downregulation of the *Adrb1* transcript levels in Tg-RKIP hearts could be attributed to the inhibition of *Grk2* by RKIP, which could prevent Grk2-mediated β1-adrenoceptor (*Adrb1*) desensitisation and downregulation.

The β1-adrenoceptor (*Adrb1*) is the predominant β-adrenoceptor in the heart and mediates catecholamine-induced inotropic and chronotropic responses in the mouse [151,152]. Notably, there is no contractility response to β-adrenoceptor agonist stimulation in *Adrb1*−/− mouse hearts, which lack the β1-adrenoceptor [151,152]. For comparison, the deficiency of the β2-adrenoceptor has little impact on cardiac performance, and physiological effects of *Adrb2* deficiency in *Adrb2*−/− hearts are only detected during exercise stress [153]. In Tg-RKIP hearts, the transcript levels of *Adrb2* were significantly increased 1.80 ± 0.27-fold compared to those of non-transgenic FVB hearts (Figure 6).

The β3-adrenoceptor (*Adrb3*) is mainly expressed in adipose tissue [151]. In agreement with these findings, the cardiac transcript levels of *Adrb3* were low in Tg-RKIP and FVB hearts (Figure 6). Nevertheless, the transgenic expression of RKIP led to a significant increase of cardiac *Adrb3* levels (Figure 6). In this respect, Tg-RKIP mice again resemble failing human hearts, which also showed upregulation of the β3-adrenoceptor [154]. Thus, transgenic RKIP expression leads to increased transcript levels of *Adrb1*, *Adrb2* and *Adrb3* in Tg-RKIP hearts.

The β1-adrenoceptor is causally involved in the development of heart failure [155,156]. Vice versa, inhibition of the β1-adrenoceptor with a prognosis-improving β1-adrenoceptor antagonist is a mainstay of human heart failure treatment [156]. The role of the β2-adrenoceptor in heart failure is less clear. In the heart, the β2-adrenoceptor transcript levels are much lower than those of the β1-adrenoceptor (Figure 6 and References [149,152]). The lower cardiac β2-adrenoceptor (*Adrb2*) levels could account for a minor role of the β2-adrenoceptor in heart failure development. The β2-adrenoceptor not only activates Gs proteins but also stimulates Gi-mediated signalling [157,158]. By activation of Gi proteins, the β2-adrenoceptor could promote cardioprotective and antiapoptotic signalling, at least at low expression levels [159,160]. In contrast, high expression levels of the β2-adrenoceptor (*Adrb2*) induce a heart failure phenotype with progressive fibrotic dilated cardiomyopathy in mice [161]. 

Acting as a GRK2 inhibitor, RKIP promotes β2-adrenoceptor sensitisation and enhances cardioprotective Gi-stimulated signalling [20]. However in vivo, these cardioprotective effects of RKIP seem to be linked to a defective *Nnt* locus, because in the presence of an intact *Nnt* locus and unrestrained mitochondrial ROS generation under cardiac stress such as pressure overload, endogenously expressed RKIP levels are sufficient to promote detrimental cardiac effects such as myocardial fibrosis [23,140]. In addition, increased RKIP expression levels are a sufficient single cause of cardiac dysfunction in Tg-RKIP mice, which finally culminates in overt heart failure symptoms with dilative cardiomyopathy, increased levels of natriuretic peptides and *Ace* and *Grk2* upregulation as indicators of an overactivated RAAS (renin angiotensin aldosterone system) and sympathetic nervous system. Therefore, the net effect of the dual RAF1 and GRK2 inhibition by RKIP in vivo is a detrimental cardiac phenotype, which predisposes to cardiac fibrosis, cardiac dilation and heart failure.

### 4.5. Summary of Heart Failure-Promoting Functions of RKIP 

**Figure 7:**
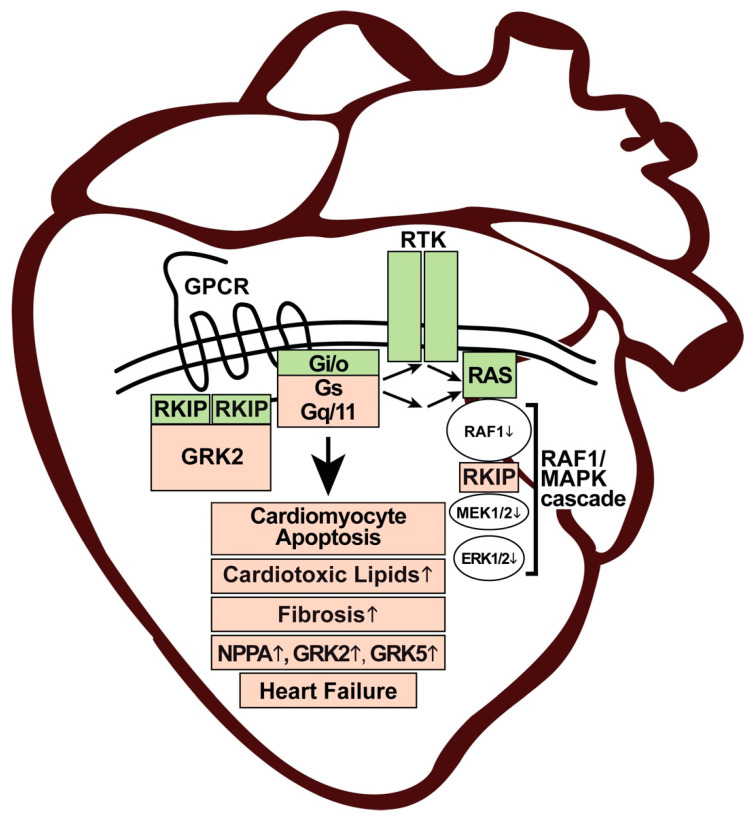
Scheme of heart failure-promoting functions of RKIP. RKIP (light red) enhances cardiomyocyte apoptosis and the accumulation of cardiotoxic lipids (light red) by inhibition of the pro-survival RAF1-MAPK cascade (white). By the inhibition of GRK2, RKIP (green) promotes beneficial GPCR-stimulated Gi/o signalling and crosstalk with RAS-stimulating receptor tyrosine kinases, RTK (green). By sensitisation of the detrimental cardiac Gs- and Gq/11-stimulated receptors, which enhance cardiac fibrosis and upregulation of NPPA, GRK2 and GRK5 (light red), RKIP promotes symptoms of heart failure. The detrimental RKIP-mediated cardiac functions are highlighted in light red (heart image adapted from: https://www.vectorstock.com/royalty-free-vector/human-heart-anatomy-organs-symbol-in-cartoon-vector-28345117?refer=eml; accessed on 26 December 2021).

This review aims to delineate how the multifunctional protein and GRK2 inhibitor RKIP accounts for a heart failure phenotype despite cardioprotective signalling stimulated by GRK2 inhibition. The cardiotoxic effects of the tumour suppressor RKIP can be attributed to inhibition of the pro-survival RAF1-MAPK pathway. By its RAF1-inhibitory function, RKIP could promote cardiomyocyte death and cardiotoxic lipid overload (Figure 7). RKIP-mediated cardiotoxic effects could further be aggravated by GRK2-inhibition-mediated sensitisation of heart failure-promoting GPCRs coupled to Gq/11 and Gs proteins (Figure 7). By sensitisation of the heart failure-promoting Gq/11-coupled AGTR1, RKIP enhances myocardial fibrosis and cardiac hypertrophy [10,21,22,23]. By sensitisation of cardiac Gs-coupled β-adrenoceptors, RKIP could promote detrimental Gs-stimulated cAMP signalling and upregulation of natriuretic peptides *NPPA* and *NPPB* [162,163]. In support of this statement, Tg-RKIP hearts with symptoms of heart failure showed upregulation of *Grk2*, which reflects sensitised β-adrenoceptor-stimulated cAMP signalling [142]. Beneficial Gi-mediated β2-adrenoceptor-stimulated signalling in mice is apparently not sufficient to overcome multiple heart deteriorating functions of RKIP in the long term [20,23]. 

By the inhibition of GRK2 and sensitisation of β-adrenoceptor-dependent Gs/Gi-mediated signalling, RKIP exerts an inotropic effect in mice [20]. In patients, approved inotropic agents are applied for the short-term enhancement of the cardiac function in acute decompensated heart failure situations [164,165,166,167]. Ideally, inotropic agents would serve as a bridge to heart transplant. However, inotropic agents have high risks and adverse effects [164,165,166,167]. Therefore, no inotrope is currently approved for long-term use in heart failure [167]. Most inotropic agents are associated with an unfavourable outcome and an increased mortality [164,165,166,167]. Adverse effects are—at least partially—attributed to an increased oxygen and energy cost of contractility [165]. In view of those available data, it is not clear whether the adverse effect profile of RKIP could be better than that of currently approved inotropes, which rely on cAMP and calcium such as beta-agonists, phosphodiesterase type-3 inhibitors and calcium sensitisers. In addition, it is not clear whether the observed RKIP-mediated sensitisation of β2-adrenoceptor Gi-coupling in rodents occurs also in humans, because failing and non-failing human hearts show a predominant coupling of β2-adrenoceptors to Gs but not Gi proteins [168]. 

Taken together, RKIP exerts cardiotoxic effects by multiple pathways (i) as a tumour metastasis suppressor and inhibitor of the RAF1-MAPK axis and (ii) as a GRK2 inhibitor (Figure 7).

## 5. Lessons from RKIP Studies on Requirements for a Cardioprotective GRK2 Inhibitor

Studies with the multifunctional GRK2 inhibitor RKIP have revealed much information on the requirements for a cardioprotective GRK2 inhibitor. Experiments with the RAF1-inhibitory RKIP documented and confirmed that an intact pro-survival RAF1-MAPK pathway is an important feature of cardioprotective GRK2 inhibition [11,21,22]. Besides inhibition of the pro-survival RAF1-MAPK pathway, RKIP exerts additional negative activities in the heart, which should be avoided in a cardioprotective GRK2 inhibitor. RKIP is a GRK2 inhibitor, which mainly interacts with the N-terminus of GRK2. By interacting with the GRK2 N-terminus, RKIP selectively inhibits the GRK2-mediated phosphorylation of GPCR substrates and sensitises GPCR-stimulated signalling (cf. Figure 2). For instance, the strong sensitisation of Gq/11-stimulated signalling by a GRK2 inhibitor could be a major concern, as was shown in different studies with RKIP [22,23]. Moreover, RKIP has no/little effect on soluble, non-GPCR substrates [10]. This lacking effect on soluble GRK2 substrates could be another negative feature of RKIP, because recent studies indicated that the inhibition of non-GPCR substrates, notable in mitochondria, is essential for the cardioprotective profile of a GRK2 inhibitor [169,170]. Usually, a kinase inhibitor is aimed to target the ATP-binding site. The antidepressant paroxetine is a low-affinity, ATP-competitive inhibitor of GRK2 with cardioprotective activities in vivo [109]. ATP-competitive GRK2 inhibitors dampen the GRK2-mediated phosphorylation of membrane-localised receptor substrates and soluble substrates. In contrast, GRK2 inhibitors such as RKIP interact with the GRK2 N-terminal domain and thereby only prevent GPCR desensitisation. By this mechanism, RKIP could be inferior to small molecule GRK2 inhibitors, which inhibit the phosphorylation of receptor and non-receptor substrates.

Despite well-documented beneficial activities, GRK2 inhibition—like all therapeutic approaches—can have adverse effects. Adverse effects of complete GRK2 inhibition are caused by the fact that GRK2 is an indispensable kinase. The homozygous *Grk2* knockout causes the lethal phenotype of *Grk2*−/− mice [96]. Therefore, highly potent inhibitors of GRK2 will have adverse effects due to blockade of indispensable GRK2 functions, e.g., GRK2 inhibitors cannot be given during pregnancy because of essential GRK2 functions during embryogenesis. In this context, proteinic GRK2 activity modulators, which disrupt protein–protein interactions such as the βARKct, with beneficial, GRK2-independent, Gβγ-scavenging activities [171], could be advantageous over highly selective, small molecule GRK2 inhibitors. Furthermore, the fine tuning of GRK2 activity to normal physiological levels is important to avoid adverse effects of too strong GRK2 inhibition.

Adverse effects of Grk2 inhibition are also detectable in adult mice. Inhibition of Grk2 in adult mice leads to weight gain under conditions of a high-fat diet intake [172]. This obesogenic phenotype has become a major concern of GRK2 inhibitors. The obesogenic phenotype of Grk2 inhibition was attributed to an increase in branched-chain amino acids (BCAA) and endocannabinoids [172]. The increase in BCAA levels upon Grk2 inhibition could be caused by decreased *Pparg*-induced *Adipoq* and *Ucp1* levels [173,174,175], which are a direct consequence of Grk2 inhibition [21]. Since increased *Adipoq* and *Ucp1* levels contribute to heart failure pathogenesis [21,122], the Grk2 inhibition-induced normalisation of pathologically elevated *Adipoq* and *Ucp1* levels is a desired, positive therapeutic effect and not an adverse effect. Moreover, by this mechanism, GRK2 inhibitors are expected to counteract symptoms of cardiac cachexia as a potentially life-threatening condition in severe heart failure [176]. The Grk2 inhibition-mediated increase in endocannabinoid levels could be attributed—at least partially—to sensitisation of Gq/11-coupled receptor signalling, e.g., stimulated by the Gq/11-coupled AGTR1 [172,177], which is known to promote weight gain [178]. As detailed above, studies with RKIP clearly documented that GRK2 inhibition-induced sensitisation of AGTR1 is detrimental [22,23]. To circumvent AGTR1 sensitisation-induced weight gain, GRK2 inhibitors could be combined with an inhibitor of the angiotensin system, i.e., an AGTR1 antagonist, an ACE inhibitor or an ARNI (angiotensin receptor neprilysin inhibitor, consisting of sacubitril and valsartan). All these angiotensin system inhibitors are prognosis-improving therapies of heart failure [156] and could counteract GRK2 inhibition-induced weight gain, which is mediated by AGTR1 sensitisation [178]. Based on the available data with paroxetine, obesogenic side effects of a GRK2 inhibitor therapy do not seem to be a major problem and/or are counteracted by co-medication with prognosis-improving cardiovascular drugs in patients [179]. As an alternative, target-specific and/or mitochondria-specific GRK2 inhibitors could be developed with good cardioprotective profile [169,170] and less side effects.

Taken together, studies with the multifunctional protein, RKIP, delineated major requirements for a cardioprotective GRK2 inhibitor. RKIP is an endogenous tumour metastasis suppressor with detrimental cardiac effects due to RAF1-MAPK pathway inhibition and its specific mode of GRK2 inhibition. Based on detrimental RKIP outcomes, future approaches of cardioprotective GRK2 inhibitor development can avoid cardiotoxic side effects and design compounds, which do not inhibit pro-survival kinases such as RAF1 but neutralise pathological GRK2 activities without disturbing indispensable physiological GRK2 functions.

What about the ongoing translational efforts of RKIP-mediated tumour metastasis suppression? The inhibition of RAF(1) kinase(s) has detrimental cardiac side effects. In this respect, RKIP resembles many approved anticancer treatment modalities, which are similarly associated with cardiovascular adverse effects [180]. However, heart failure treatment and cancer prevention do not necessarily contradict each other. Heart failure is a condition that is associated with an increased frequency of malignancies [181]. This association points to common pathways between heart failure and tumour pathogenesis. Heart failure-triggered oncogenic factors could be related to the systemically perturbed milieu of inflammatory stimuli, an increased oxidative stress and exaggerated neurohormonal activation [182,183]. In this context, more interdisciplinary research efforts between cardiologists and oncologists are needed to identify and target those pathomechanisms that link heart failure to the development of cancer.

## Data Availability

NGS data are available at the NCBI GEO Database, accession number GSE191316.
